# Anterograde Mini-Percutaneous Retropelvic Extra-Luminal Endopyelotomy: A Novel Approach to Uretero-Pelvic Junction Obstruction

**DOI:** 10.7759/cureus.22586

**Published:** 2022-02-25

**Authors:** Amadadin Alhlib, Abdullah E Laher, Ahmed Adam

**Affiliations:** 1 Urology, University of the Witwatersrand, Johannesburg, ZAF; 2 Emergency Medicine, University of the Witwatersrand, Johannesburg, ZAF

**Keywords:** extra-luminal approach, anterograde percutaneous mini-perc, endopyelotomy, pyeloplasty, upjo, uretro-pelvic junction obstruction

## Abstract

Background

Uretero-pelvic junction obstruction (UPJO) is a common cause of upper tract urinary obstruction. This condition is generally treated with various surgical options which include endoscopic (retrograde or anterograde), laparoscopic, open or robotic-assisted approaches. Herein, we describe a novel endoscopic retropelvic extra-luminal approach using a mini (14 Fr) nephroscope.

Methods

A 30-year-old male presented with symptomatic left UPJO and inferior pole renal stones, which were identified on computed tomography (CT) imaging. Mercaptuacetyltriglycine (MAG3) renogram demonstrated a functioning left kidney. With the patient positioned supine, a mini-perc (Karl-Storz) nephroscope was used to access the renal pelvis via the percutaneous route. The retropelvic space was thereafter accessed. Using a Holmium-YAG laser, the UPJO was splayed using an extra-luminal approach.

Results

Clear endoscopic vision, minimal bleeding, and overall satisfactory identification of the UPJO were achieved. At the 12-month follow-up, the patient remained stent and symptom-free. On follow-up CT imaging and MAG3 renogram, the system remained dilated with no obstruction noted.

Conclusion

Percutaneous anterograde retropelvic extra-luminal endopyelotomy is a novel approach that should be considered in patients with secondary renal calculi. This is the first report of the procedure being successfully performed utilizing the mini-perc access route.

## Introduction

Uretero-pelvic junction obstruction (UPJO), which can be described as primary or secondary, is one of the commonest causes of hydronephrosis. It is commonly associated with other concurrent pathologies such as nephrolithiasis or pelvicalyceal urothelial tumors [1].

Several approaches, including endoscopic (retrograde or anterograde), laparoscopic, open or robotic-assisted, have been described in the management of UPJO [1]. However, the selection of the optimal approach can be challenging. There is controversy in terms of which of these approaches is superior and associated with lower re-operative and complication rates.

Endoscopic endopyelotomy is described to have fewer complications than laparoscopic, open, or robotic-assisted approaches. The obstruction may be accessed either via a ureteroscopic retrograde approach or a nephroscopic percutaneous anterograde approach [1].

The anterograde retropelvic extra-liminal endopyelotomy approach allows for the stricture to be visualized and accessed from the external ureteral wall, while with the standard endopyelotomy technique, the stricture is incised from the inner wall (luminal) side [2]. Although this procedure has been described using the maxi/standard nephroscope. Herein we describe the novel endoscopic retropelvic approach using a mini (14 Fr) nephroscope.

Ethics approval was obtained from the Human Research Ethics Committee of the University of the Witwatersrand (certificate no. M1911178). The abstract was presented at the 30th Malaysian Urological Conference that was held on November 26 and 27, 2021 and was published in BJU International [3].

## Materials and methods

**Figure 1:**
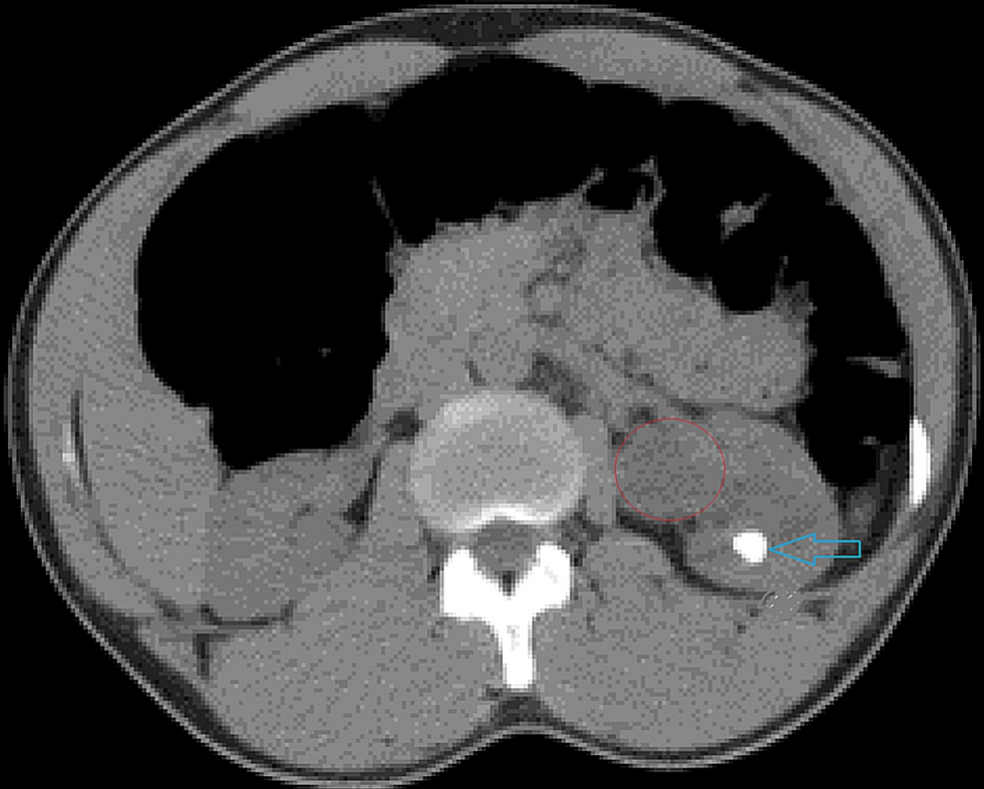
Non-contrasted CT scan showing left-sided 12.7 mm x 9.8 mm renal calculus (blue arrow) and dilated uretero-pelvic junction segment (red circle).

A 30-year-old male with a body mass index of 29 kg/m^2^ presented to the emergency department with left flank pain. He had no prior history of renal or ureteric calculi, nor was there any other significant past medical or surgical history of note. On further investigation, he was noted to have a normal renal function and no elevation of septic markers. Radiological imaging (computed tomography [CT] scan) revealed left-sided hydronephrosis with two discrete renal calculi (12.7 mm x 9.8 mm [Figure 1] and 6.9 mm x 4.7 mm) and a 2-mm left ureteric calculus fragment (which subsequently passed spontaneously).

Mercaptuacetyltriglycine (MAG3) renogram demonstrated a split function of 62% of the left kidney and 38% of the right kidney with an obstructive curve pattern. A mini-percutaneous nephrolithotomy (PCNL) and anterograde retropelvic extra-luminal endopyelotomy was performed six-weeks after the initial presentation. The patient was not prestented prior to the procedure.

The procedure

**Figure 2:**
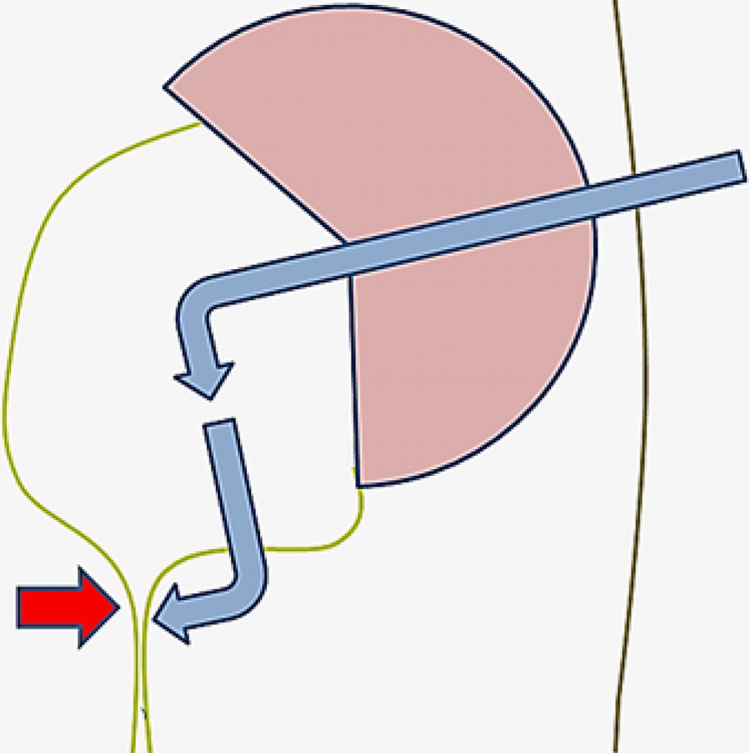
Schematic diagram of left kidney illustrating the approach to the retropelvic space using the conventional percutaneous renal access method. The red arrow highlights the anatomical region of the UPJO, which is approached via the retropelvic route. The blue arrows represent the access method into the kidney via the renal pelvis and the extra-luminal access route utilized to incise the UPJO from the retropelvic space. This approach allows for better visualization of the UPJO segment, resulting in a more controlled laser incision over the ureteric luminal wall from outside the ureter.

**Figure 3:**
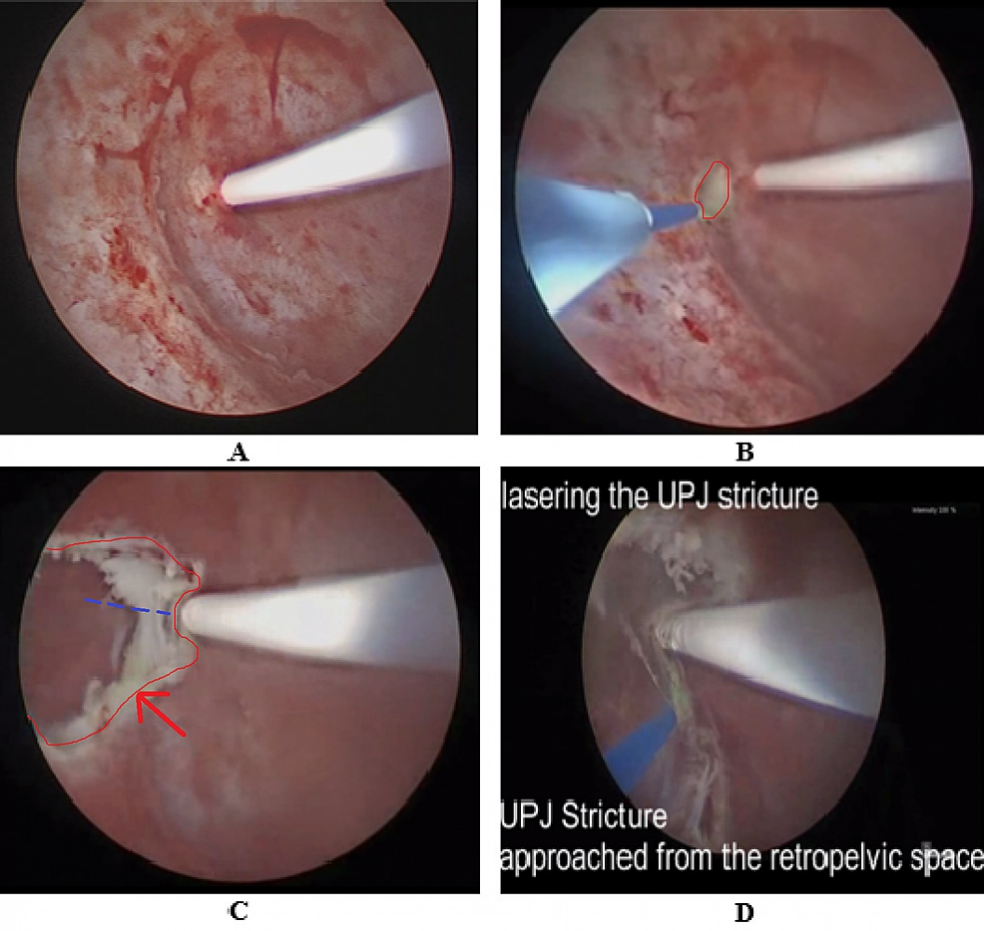
(A) Endoscopic view of the UPJO. The ureteric catheter is seen entering the renal pelvis. (B) Laser incision was performed lateral to the UPJO segment to create a window of access to the retropelvic space (red circle demarcates the opening). (C) Opening into the retropelvic space was expanded (outlined in red). The cleavage plane was opened (parallel to the ureter) using the nephroscope sheath (red arrow). This allowed lateral extra-luminal retropelvic access to the ureter. Laser incision was thereafter performed on the extra-luminal surface of the ureter (dashed blue line). (D) The ureteric stent was well demarcated and used as a marker to guide the incision depth. This allowed for an adequate incision of the stenotic UPJO segment. UPJO - Uretero-pelvic junction obstruction

The patient was placed in a supine position. Retrograde pyelogram was performed and an open-ended size 6 Fr ureteric catheter was advanced up to the renal pelvis. Via anterograde percutaneous access, a 14 Fr nephroscope was used to extract both renal calculi. Thereafter, a 365-micron holmium-YAG laser fiber was advanced through the scope to perform a full-thickness pelvic wall cut outlining an imaginary line between the UPJ and the lower pole pelvicalyceal junction until the retropelvic fat was visualized. Thereafter a plane was created with the nephroscope to provide lateral retropelvic access to the extra-luminal UPJO segment so as to facilitate an extra-luminal controlled laser incision. Using the ureteric stent as a guide to the inner limit of incision, the incision was carried out from the extra-luminal retropelvic space on the external surface of the ureter (Figures 2, 3).

Clear vision and homeostasis were maintained throughout the procedure. A ureteral stent (26 cm/6 Fr) was thereafter advanced into the bladder in an anterograde fashion and a nephrostomy tube was successfully placed. Total operative time was 50 minutes and the patient was discharged from the hospital on the second postoperative day.

## Results

The patient’s postoperative course was unremarkable. The ureteral stent was subsequently removed, with the retrograde study showing no residual UPJ stricture.

At the 12-month follow-up, the patient remained stent and symptoms-free. A repeat MAG3 scan demonstrated the split function of 57% and 43% of the left and right kidneys, respectively, and delayed excretion on the left side with no obstruction.

## Discussion

Endopyelotomy may be performed using either a retrograde or anterograde approach. Each has distinct advantages and complications; however, both procedures have been associated with shorter hospital stays and less operating room time than the open or laparoscopic pyeloplasty procedures [4,5].

The procedure entails a full-thickness incision of the UPJO under endoscopic or fluoroscopic guidance, using either a cold knife, diathermy, or laser fiber. Allowing the UPJ segment to heal around a ureteral stent may result in a wider diameter, with an average success rate of 82% (73%-90%) [6-8]. The healing response is also dependent on the length of the obstructing segment, the severity of hydronephrosis, recurrence after previous repair and the presence of a crossing vessel [5]. In contrast, the open or laparoscopic approaches have been associated with success rates of 90%-100% [9,10].

The novel anterograde retropelvic endopyelotomy was first described by Khalid Alotaibi, who performed the procedure on 39 patients in the supine position, using the Lawson retrograde nephrostomy wire puncture to access the collecting system. The study showed promising results, with a success rate of 90% in general. The procedure success rate was only affected by low kidney split function (<35%) on the MAG3 renogram [2].

Anterograde endopyelotomy is the procedure of choice for UPJ obstruction associated with urolithiasis. Since both conditions can be managed simultaneously, positioning the patient in the supine position allows for both retrograde and anterograde access and may also be associated with fewer anesthetic challenges compared to the prone position [11,12]. In our patient, we used a one-step dilatation technique which is associated with less bleeding and a lower probability of requiring a blood transfusion [13]. Endopyelotomy of the renal pelvis and UPJ was performed using a Holmium-YAG laser which is advantageous in that it has both tissue cutting and homeostasis capability compared to endoscopic cold knife or balloon dilation.

Compared to the laparoscopic approach, the endoscopic approach has been associated with shorter operative times, however, the laparoscopic approach has been associated with better success rates (100% vs 92.8%) [1]. Pardilidis et al., have recommended percutaneous endopyelotomy as the treatment of choice for intrinsic UPJO and laparoscopic dismembered pyeloplasty as the treatment of choice for extrinsic UPJO [4].

In cases of UPJO, selection of the optimal surgical approach is dependent on anatomical considerations, prior surgery, patient expectation and the attending surgeon’s experience [14]. Since the retropelvic endopyelotomy approach is associated with success rates of 100% [2], we opted for this technique in our patient. Regarding the role of the anterograde retropelvic endopyelotomy in cases of UPJO, a previous series has shown promising results. This positive outcome was observed to be more pronounced in patients with associated renal calculi and good renal split function (>35%) [1]. Contraindications to this technique are the same as for all percutaneous renal surgery procedures and include bleeding disorders, untreated urinary tract infections and pregnancy [15].

The benefits of using a smaller caliber nephroscope compared to a standard sheath (in gaining access in this technique) have been proven to be numerous and include less time required to achieve percutaneous access, lower bleeding risk, less post-operative pain and shorter hospital stay [16].

An obvious limitation included within this paper is the absence of a large dataset of patients. However, since this was a novel approach to the retropelvic access of UPJO, the viability of the current technique is the core of what was presented.

## Conclusions

We have described a novel technique using a less invasive mini-percutaneous access sheath to perform anterograde extra-luminal endopyelotomy, via the retropelvic space. We have demonstrated good visualization and minimal bleeding, all while achieving the desired operative objectives. This approach allows for an extra-luminal incision of the UPJO segment in a controlled fashion over the ureteric stent. The stent acts as the intra-luminal incision limit. This procedure can be regarded as an additional option in the armamentarium of UPJO management for the attending urologist.
